# Experimental test of the fitness effects of divergent marine–freshwater chromosomal inversions in stickleback under different salinity conditions

**DOI:** 10.1038/s41437-025-00784-8

**Published:** 2025-07-24

**Authors:** Juliana Rodríguez-Fuentes, Nicole Nesvadba, Verena Saladin, Marius Roesti, Catherine L. Peichel

**Affiliations:** Division of Evolutionary Ecology, Institute of Ecology and Evolution, University of Bern, Bern, Switzerland

**Keywords:** Evolutionary genetics, Genetic linkage study

## Abstract

Chromosomal inversions are a type of structural variant that have long interested evolutionary biologists because of their potential role in local adaptation and speciation. However, direct experimental evidence for the fitness consequences of inversions is rare, limiting our ability to dissect the evolutionary forces associated with the spread and maintenance of inversions in natural populations. We tackle this knowledge gap by studying the fitness effects of three chromosomal inversions that consistently differ between marine and freshwater populations of threespine sticklebacks (*Gasterosteus aculeatus*). Using controlled laboratory crosses, we tested whether inversion genotype influences fitness (measured as survival, standard length, and body condition) across two salinity treatments (freshwater vs saltwater). In both the freshwater and the saltwater treatments, there were no deviations from Mendelian ratios at any of the three inversions. This suggests that there are no intrinsic deleterious effects of these inversions, in contrast to observations from other systems. Overall, there was no effect of inversion genotype on standard length or body size across the two salinity treatments for the chromosome XI and XXI inversions. For the chromosome I inversion, heterozygotes had a slightly lower body condition in the freshwater treatment. Together, these results suggest that the fitness effects of these inversions are not strongly influenced by salinity and that other selective forces might be involved in their evolution. More broadly, these findings highlight the importance of performing empirical tests of fitness effects of chromosomal inversions to better explain their spread and maintenance in nature.

## Introduction

Inversions are a type of structural variant in which the orientation of a chromosomal segment is reversed relative to the “standard” orientation. These chromosomal changes have long interested evolutionary biologists, mainly due to their effect on recombination (Kirkpatrick 2010; Wellenreuther and Bernatchez 2018; Faria et al. 2019). When an individual is heterozygous for the standard and the inverted orientation, crossovers within the inverted segment produce inviable gametes (Hoffmann and Rieseberg 2008; Kirkpatrick 2010). Thus, there is no effective recombination in inversion heterozygotes, except when there are double crossovers or gene conversion events (Navarro et al. 1997). As recombination modifiers, inversions are proposed to aid in adaptation and speciation because they facilitate the independent evolution of the genomic regions where they occurred, which allows the accumulation of adaptive alleles without the homogenizing effect of recombination (Kirkpatrick and Barton 2006; Hoffmann and Rieseberg 2008; Kirkpatrick 2010; Wellenreuther and Bernatchez 2018; Faria et al. 2019). Consistent with this theoretical rationale and aided by advances in genome sequencing, chromosomal inversions have increasingly been found in studies of genomic divergence between ecotypes or closely-related species (Lowry and Willis 2010; Wellenreuther and Bernatchez 2018; Koch et al. 2022; Hager et al. 2022). However, direct experimental tests of the fitness effects of inversions remain scarce, limiting our understanding of the evolutionary significance of inversions and the forces shaping their frequency in nature (Kirkpatrick 2010; Wellenreuther and Bernatchez 2018; Faria et al. 2019).

Indeed, the establishment and persistence of inversions in nature are likely shaped by multiple processes, making it challenging to distinguish between alternative explanations for patterns observed in the wild. Initial establishment is limited because inversions have low starting frequencies and can be easily lost by drift, with selection increasing the likelihood of loss if inversions cause deleterious effects (Faria et al. 2019). Underdominance, or the lower fitness of heterozygotes due to meiotic problems, is a commonly proposed deleterious effect. Although evidence suggests that this is mostly common in plants, a systematic evaluation of the underdominant effects of inversions is lacking (Hoffmann and Rieseberg 2008; Berdan et al. 2023). Despite their low starting frequency and potentially underdominant effects, inversions can spread in a population by drift or selection (Kirkpatrick and Barton 2006; Hoffmann and Rieseberg 2008; Faria et al. 2019). The spread of an inversion is determined by standard population genetic parameters, of which the fitness variance per unit map length is a key factor determining the likelihood and speed of establishment (Berdan et al. 2023). In scenarios where selection is involved, inversions can be established through direct or indirect effects on fitness (Faria et al. 2019). Hypotheses based on direct selection propose that the mutation created by the inversion itself has a selective advantage, thus favoring its spread. Such a mutation can disrupt a gene or modify its expression (Hoffmann and Rieseberg 2008; Kirkpatrick 2010; Guerrero et al. 2012; Villoutreix et al. 2021), but empirical evidence for direct beneficial effects of mutations created by inversions is limited to a study in *Timema* stick insects (Villoutreix et al. 2020). Thus, additional mechanisms are likely to explain the spread of inversions in most systems.

The spread of inversions due to indirect selection is likely applicable under a broader range of biological conditions. Some hypotheses invoke the effect of inversions on recombination because they can hold together adaptive alleles that are beneficial in specific environments, either due to epistatic selection on co-adapted alleles (Dobzhansky 1970) or due to independent selection on locally-adapted alleles (Kirkpatrick and Barton 2006). Alternative hypotheses invoke mechanisms such as negative frequency-dependent selection or higher fitness of heterozygous (overdominance) to explain balanced polymorphic inversions (Hoffmann and Rieseberg 2008; Berdan et al. 2023). Overdominance can result from the effect of individual loci within the inversion, or the linkage between neutral loci and recessive deleterious or dominant beneficial mutations, known as associative overdominance (Faria et al. 2019; Durmaz et al. 2020). Indeed, linkage between neutral loci to sites under negative selection is facilitated within inversions because they accumulate recessive deleterious mutations due to the reduced recombination in these regions (Ohta 1971; Berdan et al. 2021). Empirical evidence for the accumulation of recessive deleterious mutations in inversions has been found in seaweed flies (*Coelopa frigida*) (Butlin and Day 1985; Mérot et al. 2020), *Drosophila melanogaster* (Kapun and Flatt 2019)*, Heliconius* butterflies (Jay et al. 2021), and zebra finches (*Taeniopygia guttata*) (Knief et al. 2017). A common element to these hypotheses is the effect that inversions have on fitness, but relatively few studies have directly estimated fitness effects of inversions (Lowry and Willis 2010; Ayala et al. 2014; Lee et al. 2016; Kapun et al. 2016; Knief et al. 2016; Kapun and Flatt 2019; Mérot et al. 2020; Jay et al. 2021; Pei et al. 2023; Nosil et al. 2023). Thus, to shed more light on the evolutionary forces acting on inversions, it is crucial to estimate their fitness effects in natural systems.

A well-suited system for studying the fitness effects of inversions is the threespine stickleback (*Gasterosteus aculeatus*). This fish species colonized freshwater habitats from a marine ancestor multiple times after the last glacial maximum (Bell and Foster 1994; McKinnon and Rundle 2002), which was facilitated by the reuse of shared ancestral haplotypes that have been maintained in the marine population (Bassham et al. 2018; Colosimo et al. 2005; Jones, Grabherr, et al. 2012; Nelson and Cresko 2018). Among these haplotypes, three paracentric chromosomal inversions, located on chromosomes (chr) I, XI, and XXI, are consistently divergent between marine and freshwater populations (Fang et al. 2020; Jones, Grabherr, et al. 2012; Magalhaes et al. 2021; Roberts Kingman et al. 2021). In these populations, the marine and freshwater homozygotes are at high frequencies in their respective environment (Liu et al. 2018), suggesting no strong deleterious effects of the inversions in these environments. However, this has not been empirically tested. Moreover, these inversions have also been found to be divergent between freshwater ecotypes (Jones, Chan, et al. 2012; Roesti et al. 2015; Haenel et al. 2019), but little is known about the mechanisms driving these associations.

Despite the limited knowledge about these inversions in sticklebacks, preliminary evidence suggests potential sources of selection acting on them. The chromosome I inversion contains the Na(+)/K(+) transporting ATPase gene (*Atp1a1a*), which is generally involved in fish osmoregulation (Evans et al. 2005). This gene shows repeated genomic (Jones, Grabherr, et al. 2012; Magalhaes et al. 2021) and expression (Verta and Jones 2019) divergence between multiple pairs of marine and freshwater populations and signatures of directional selection associated with environmental salinity (Shimada et al. 2011). These results suggest that salinity could be a selective pressure acting on this inversion. In the chromosome XI inversion, the voltage-gated potassium channel gene *KCNH4a* is located across one of the breakpoints, and it has been suggested that the inversion creates different marine and freshwater transcripts due to the presence of duplicated 3’ exons outside this inversion breakpoint (Jones, Grabherr, et al. 2012). Taugbøl and collaborators (2014) found higher expression of *KCNH4a* in the gills of freshwater sticklebacks. However, they did not determine the inversion genotype at chromosome XI, and it is therefore unclear whether the freshwater orientation leads to this higher expression. The chromosome XXI inversion is a hotspot of quantitative trait loci (QTL) associated with a range of morphological traits that diverge between ecotypes (Peichel and Marques 2017), which indicates the possibility that multiple adaptive loci are held together in this inversion as predicted by the local adaptation model (Kirkpatrick and Barton 2006). Although these studies suggest potential selective targets associated with these inversions, formal tests for these hypotheses and an assessment of the fitness consequences of these inversions are needed.

**Fig. 1:**
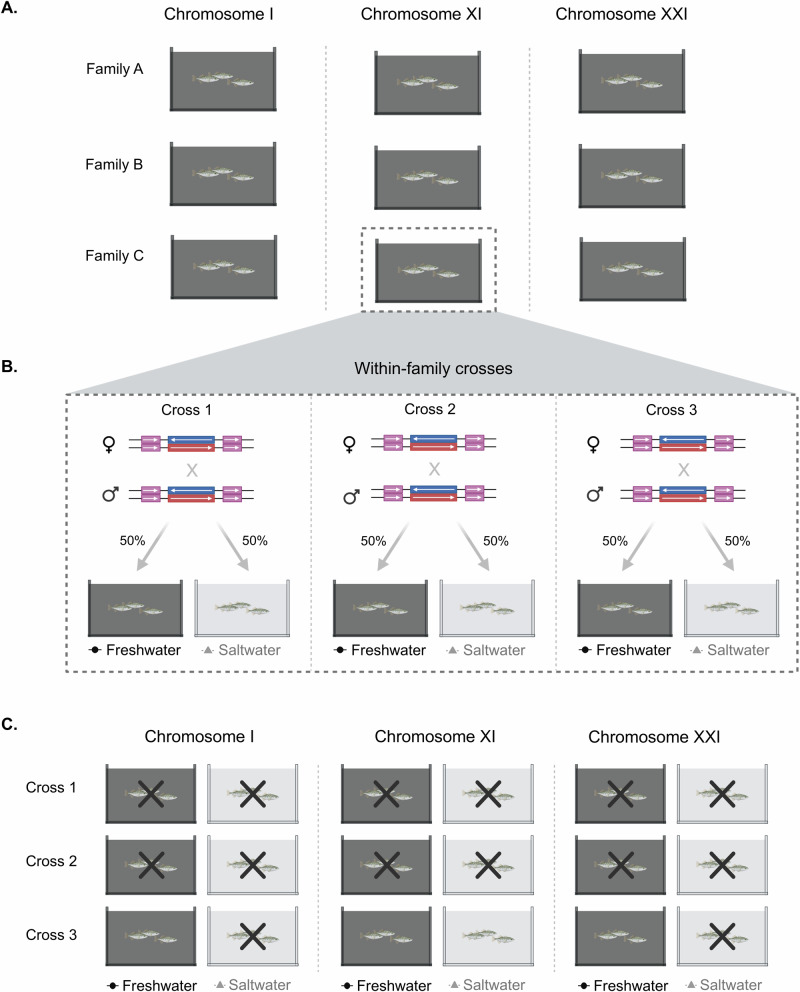
Study design to test the fitness effects of chromosomal inversions in two salinity treatments. **A** For each chromosomal inversion, three independent F1 families were generated. **B** For the F2 generation, three crosses were generated from three unique pairs of females and males taken from each independent F1 family, for a total of nine crosses per inversion. The progeny of each cross was equally divided between the freshwater and saltwater treatments. **C** The three crosses for one family per inversion are shown. For the inversion on chromosome XI, two crosses with its respective tanks in freshwater and saltwater were sampled, whereas for the inversions on chromosome I and XXI, two crosses plus the saltwater tank from the third cross were sampled. An X indicates the crosses that were sampled. This sampling procedure was equally done across the three independent families for each chromosomal inversion.

To tackle this knowledge gap, we investigated whether the chromosomal inversions in threespine sticklebacks have strong fitness effects under laboratory conditions. To do so, we made crosses between marine-derived individuals that were heterozygous for the marine and the freshwater orientations at each of the three inversions, while standardizing for a common marine background (Fig. 1). Given their strong association to marine–freshwater divergence, we explored whether variation in fitness is associated with salinity. Therefore, offspring were divided between freshwater and saltwater treatments to assess whether inversion genotype, salinity treatment, and/or the interaction between inversion genotype and salinity treatment influences three proxies of fitness: survival, standard length, and body condition. We predicted that fitness would differ among genotypes and that these differences would depend on the salinity treatment. Specifically, we predicted homozygous marine individuals would have higher fitness in the saltwater treatment, while homozygous freshwater individuals would have higher fitness in the freshwater treatment.

## Materials and Methods

### Sample collection and experimental design

Wild sticklebacks were collected from Oyster Lagoon, British Columbia, Canada (49°36'43.4“N 124°01'52.3”W) in the spring of 2022. At the phenotypic and genomic level, this is a marine population, but heterozygous individuals carrying the marine and freshwater inversion haplotypes occur at low frequency (Barrett et al. 2008; Miller et al. 2019). Wild-caught fish were captured using minnow traps and genotyped for the three inversions studied here (i.e., chromosome I, chromosome XI, and chromosome XXI, see Inversion and sex genotyping).

Individuals that were heterozygous at one of the inversions and homozygous marine at the other two inversions were used to produce F1 families. In total, we made nine F1 families (three families for chromosome I, three families for chromosome XI, and three families for chromosome XXI; Fig. 1A). Each F1 family had a unique mother and a unique father. The F1s were grown to adulthood and genotyped to identify individuals that were heterozygous at one of the inversions and homozygous marine for the other two (see Inversion and sex genotyping). These F1 heterozygous individuals derived from three independent F1 families were used to generate F2 crosses. Within each of the three F1 families, we made three replicate crosses using a different pair of F1 heterozygotes (Fig. 1B). Thus, we made a total of 27 F2 crosses (nine crosses per inversion). The F2 offspring from each cross were split equally and raised in either saltwater or freshwater until between 9- and 11-months post-fertilization. At this point, we sampled the individuals from the freshwater and saltwater treatments from two of the three replicate crosses for all chromosomes, and the saltwater treatment from the third replicate cross for chromosomes I and XXI (Fig. 1C). The remaining replicate crosses were used for a different experiment and therefore not reported here. Fish were euthanized, genotyped, and measured (standard length and weight) to assess fitness via survival, standard length, and body condition.

### Generation of F1 and F2 crosses

For all crosses, gravid females were gently squeezed to release their eggs into a Petri dish. Reproductive males were euthanized using a lethal dose of buffered MS-222 (0.2% tricaine methane sulfonate). Testes were removed from euthanized males and placed in a Petri dish containing the eggs. Fertilization was ensured by observing the eggs under a dissecting microscope.

For the F1 families, fertilized eggs were kept at 3 parts-per-thousand (ppt) saltwater for two days at 17 °C, then placed into 50 mL conical tubes with 3 ppt saltwater just covering the eggs and shipped overnight at 4 °C to the University of Bern stickleback facility. Fertilized eggs were placed in 3 ppt saltwater at 16 °C. After hatching, the resulting F1 individuals were housed in freshwater conditions at 3 ppt saltwater, grown to reproductive maturity, and then genotyped to identify heterozygous individuals at the inversion of interest (see Inversion and sex genotyping).

For the F2 crosses, the fertilized eggs were placed in a Petri dish containing a 3 ppt saltwater solution, which was changed every two days, in a cooled incubator at 15 °C. After hatching at 10–12 days post-fertilization, larvae were counted and divided equally between freshwater or saltwater treatments. The larvae in the freshwater treatment were maintained in a concentration of 3 ppt saltwater with daily water changes. The larvae in the saltwater treatment were gradually acclimatized to a concentration of 30 ppt saltwater by 10 ppt steps that started from a 10 ppt saltwater solution on the day the larvae were divided into treatment groups. Three days later, hatchlings were released into 100-L tanks with the corresponding saltwater concentrations, 3 ppt (freshwater treatment) and 30 ppt (saltwater treatment).

### Fish housing and care

Individuals from F1 and F2 crosses were housed in 100-L tanks on a recirculating system with controlled pH (~8.0) and water temperature (~16.0 °C). Salinity (measured as conductivity) was controlled to a value of approximately 5.3 millisiemens/cm (3 ppt) for the freshwater system and 46 sillisiemens/cm (30 ppt) for the saltwater system, using Instant Ocean sea salt (Instant Ocean, Aquarium Systems, Sarrebourg, France). Lighting was programmed with 14 h full sunlight (3450 lumens), 1 h sunrise, 1 h sunset, and a moonlight (600 lumens) for nighttime. Fry and juveniles were fed brine shrimp nauplii twice per day, and adult fish were fed brine shrimp in the mornings and frozen Mysis shrimp two times per week in the afternoon. The volume of food for F2 crosses was standardized to provide the same amount of food in the freshwater and saltwater treatments.

### Sampling and measuring fitness proxies of F2 individuals

The sampling consisted of euthanizing all individuals from a tank with a lethal dose of buffered MS-222 (0.2% tricaine methane sulfonate). Each fish was blotted dry, and the right pectoral fin was stored in 100% ethanol for further genetic analyses. We measured the standard length (SL), described as the length from the most anterior point of the snout to the attachment of the caudal fin, using manual calipers, and the weight using a digital scale with a precision of 0.01 mg.

### Inversion and sex genotyping

Individuals were genotyped for the three inversions and sex with PCR. Genotyping of the inversions is based on the sequence divergence between the marine and freshwater haplotype at the three inversions. Multiple sequences were obtained across the global distribution of sticklebacks, and marine and freshwater populations were compared to detect variants with an *F*_ST_ of 1 in the regions that contain the inversions. Diagnostic indels within each inversion region were chosen to differentiate the marine and freshwater haplotypes. Primers flanking the indel were designed and tested in individuals with known inversion genotypes (Table S1). To obtain the sex of individuals, we used the marker LRR (Archambeault et al. 2020), which is based on an indel that differs between the X and Y chromosomes (Table S1).

DNA was extracted using a modified HotSHOT DNA extraction method (Meeker et al. 2007; Archambeault et al. 2020) from fresh caudal fin clips of wild-caught and F1 individuals or ethanol-stored pectoral fins of F2 individuals. We did PCR in a 5 µL volume that consisted of 2.5 µL GoTaq (Promega, GoTaq® G2 Hot Start Green Master Mix), 1 µL of water, 0.5 µL10 µM reverse and forward primers, and 0.5 µL of DNA. Inversion genotyping used the following cycling protocol: an initial denaturation step at 95 °C for 1:30 min, followed by 5 cycles of denaturation at 94 °C for 30 s, annealing at 56 °C for 30 s, extension at 72 °C for 30 s, which then was followed with 30 cycles of denaturation at 90 °C for 30 s, annealing at 56 °C for 30 s, extension at 72 °C for 30 s, this was followed with a final extension step at 72 °C for 5 min. Sex genotyping used the following cycling conditions: an initial denaturation step at 94 °C for 1 min, followed by 5 cycles of denaturation at 94 °C for 20 s, annealing at 65 °C for 20 s, extension at 72 °C for 1 min, which then was followed with 26 cycles of denaturation at 94 °C for 20 s, annealing at 57 °C for 20 s, extension at 72 °C for 1 min, this was followed with a final extension step at 72 °C for 5 min.

### Survival analysis

Differences in survival were assessed between treatments and among genotypes. We calculated the survival proportion as 1 minus the difference between the number of larvae placed in the tank and the number of surviving fish at the end of the experiment, divided by the number of larvae placed in the tank. The effect of salinity treatment on survival was tested using a linear model, with treatment, family, and age as independent variables. Age, which refers to the number of days from the start to the end of the experiment, was included as part of the model because the crosses were sampled at slightly different ages which could affect our survival observations. We also assessed whether the genotype frequencies differed between treatments. For each family, we pooled the independent replicate crosses within a family and compared the genotype frequencies between the freshwater and saltwater treatments using a chi-squared test. For the chromosome I and XXI inversions, we removed the replicate cross that was sampled in the saltwater but not the freshwater treatment from the analysis.

To test whether there are differences in survival within a single generation among the individuals with different inversion genotypes, we compared the observed genotype frequencies versus the expected Mendelian ratio using a chi-squared test. As for the comparison between treatments, we pooled the observations from the independent crosses within a family. However, here, we compared the expected and observed genotype frequencies separately for the freshwater and saltwater treatments.

### Analysis of standard length and body condition

To assess if standard length and body condition are associated with genotype at the inversion, treatment or its interaction, we used linear mixed models. For each chromosomal inversion, we had two models, one for standard length and another for body condition. Body condition was calculated as the residuals from the linear regression of the cube root of the weight on standard length. These residuals were used as the dependent variable and are hereafter referred to “body condition”. This measurement serves as a proxy of an individual’s energetic status, with higher values indicating relatively greater mass for a given size, and thus greater energy reserves. In threespine sticklebacks, this metric has been associated with greater reproductive capacity (Bagamian et al. 2004). In both models, we used genotype, treatment, the interaction between genotype and treatment, sex, and age as fixed factors, whereas the source F1 family was included as a random factor. In some crosses, we observed that some individuals presented developmental abnormalities (e.g., shorter or bent vertebral columns), which were recorded at the time of euthanasia. These individuals had a higher body condition when compared to individuals from the same tank with a similar standard length and were removed from the analyses.

## Results

### Survival is not associated with inversion genotypes for either salinity treatment

Across all inversions, genotypes, and treatments, survival ranged between 20% to 100% across tanks, with a mean survival rate of 70.1 ± 19.6% in the freshwater treatment and 74.5 ± 20.9% in the saltwater treatment. Salinity treatment did not influence observed survival (F_1,31_ = 1.27, *p* = 0.27). Among the covariates, there was no effect of age on survival (F_1,31_ = 0.47, *p* = 0.49), but there was a significant effect of family (F_8,31_ = 9.52, *p* < 0.0001).

**Fig. 2:**
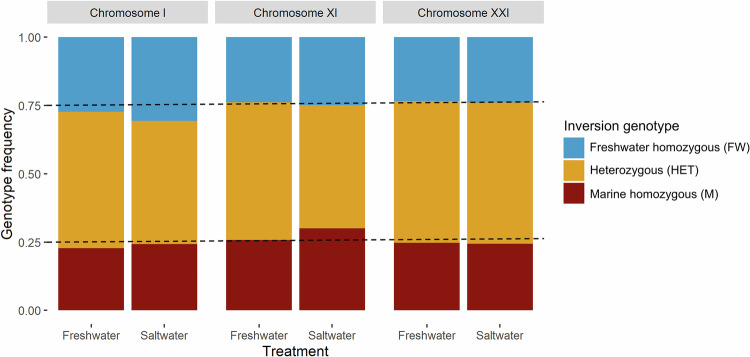
Inversion genotype frequencies at the end of the experiment. The genotype frequencies for the inversions on chromosomes I, XI, and XXI across the two experimental treatments (freshwater vs saltwater) are shown. The dotted lines at 0.25 and 0.75 indicate the expectation for Mendelian frequencies. The data shown here represent the pooled data across the three replicate families for each inversion. Sample sizes and statistical analyses are shown in Table 1.

Importantly, we found no differences in genotype frequencies between treatments for any of the three chromosomal inversions (Table 1, Fig. 2). Additionally, genotype frequencies within treatments did not deviate from the expected Mendelian ratios at any of the three chromosomal inversions (Table 1, Fig. 2).

**Table 1:** Effect of inversion genotype on survival between and within salinity treatments.

Chromosomal inversion	Family	Comparison between treatments			Comparison to the Mendelian expectation					
					Freshwater treatment			Saltwater treatment		
		*n*	X^2^	*p*-value	n	X^2^	*p*-value	*n*	X^2^	*p*-value
Chromosome I	chrI_03	102	0.65	0.70	52	0.12	0.94	95	3.82	0.15
	chrI_04	118	1.77	0.44	60	0.10	0.95	91	5.90	0.06
	chrI_06	88	5.26	0.076	42	1.58	0.46	78	3.84	0.15
Chromosome XI	chrXI_01	86	3.48	0.20	42	1.81	0.41	44	1.64	0.44
	chrXI_04	181	0.28	0.87	79	0.75	0.69	102	2.37	0.31
	chrXI_05	61	2.92	0.26	34	1.06	0.59	27	2.11	0.35
Chromosome XXI	chrXXI_04	100	5.28	0.06	50	3.88	0.14	88	2.25	0.32
	chrXXI_07	53	1.22	0.61	28	1.14	0.56	44	1.09	0.58
	chrXXI_08	154	1.75	0.41	76	0.71	0.70	106	1.62	0.44

Genotype frequencies of fish surviving until the end of the experiment do not differ between the freshwater and saltwater treatments and do not deviate from the Mendelian expectation within treatments. All results shown are from the chi-squared test using the within-family pooled data. For a visual representation of this data, see Fig. 2.

### Inversion genotype and its interaction with treatment do not influence body size

**Fig. 3:**
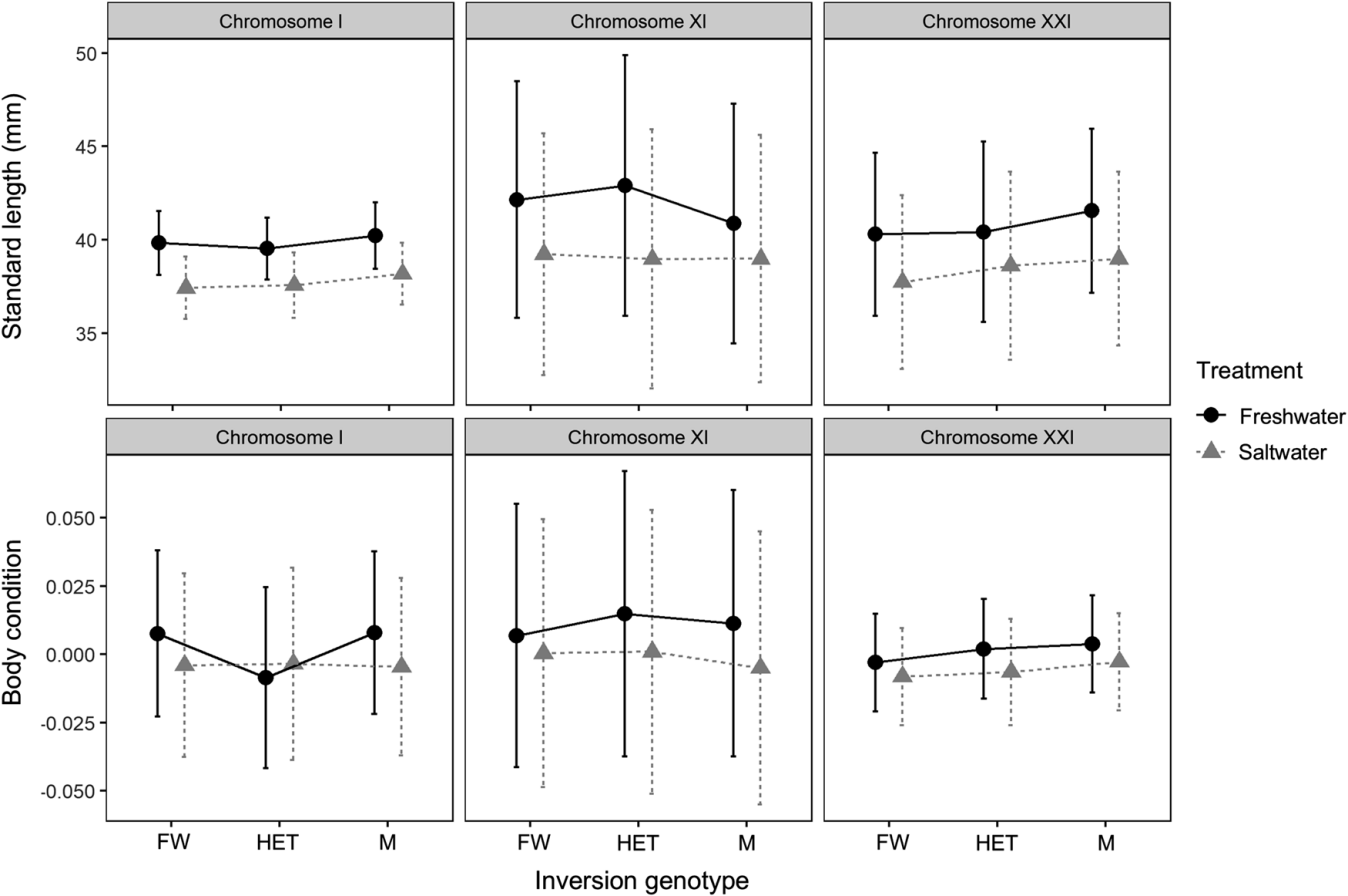
Standard length and body condition across inversion genotypes and salinity treatments. The estimated marginal means and 95% confidence intervals for standard length and body condition from linear mixed models are shown across the three chromosomal inversions. FW denotes individuals homozygous for the freshwater-prevalent inversion orientation, HET denotes heterozygous individuals, and M denotes individuals homozygous for the marine-prevalent inversion orientation. Data are jittered within genotype classes to better show the confidence intervals for each treatment. These results are based on the data across the three replicate families for each inversion. Sample sizes and statistical analyses are shown in Table 2. The effects of sex are shown in Fig. S1.

Body size, measured as standard length, was not influenced by inversion genotype or its interaction with treatment for any of the three inversions (Table 2). However, across families segregating for each of the three chromosomal inversions, treatment, sex, and age influenced standard length (Table 2). Individuals in the freshwater treatment (Fig. 3), males (Fig. S1), and older individuals were always longer.

**Table 2:** Results of model testing the effect of inversion genotype and salinity treatment on the body size measurements.

Chromosomal inversion	Predictor	Standard length				Body condition			
		D.F	Sum. Sq	F-value	*p*-value	D.F	Sum. Sq	F-value	*p*-value
Chromosome I *N* = 406	Genotype	2, 396	24.96	0.88	0.41	2, 396	0.0055	2.81	0.062
	Treatment	1, 398	378.56	26.59	**<0.001**	1, 397	0.0033	3.41	0.065
	Sex	1, 397	72.23	5.07	**0.02**	1, 396	0.022	22.25	**<0.001**
	Age	1, 164	880.06	61.81	**<0.001**	1, 379	0.067	69.09	**<0.001**
	Genotype x Treatment	2, 397	3.42	0.12	0.88	2, 396	0.0067	3.47	**0.032**
Chromosome XI *N* = 327	Genotype	2, 317	58.10	1.19	0.31	2, 317	0.0017	0.90	0.41
	Treatment	1, 317	626.52	25.61	**<0.001**	1, 317	0.011	11.93	**<0.001**
	Sex	1, 317	171.60	7.01	**0.0085**	1, 317	0.051	55.74	**<0.001**
	Age	1, 319	267.11	10.92	**0.0011**	1, 319	0.0086	9.35	**0.0024**
	Genotype x Treatment	2, 317	60.11	1.23	0.29	2, 317	0.0011	0.60	0.54
Chromosome XXI *N* = 384	Genotype	2, 374	69.86	2.33	0.099	2, 374	0.0016	0.73	0.48
	Treatment	1, 374	422.30	28.13	**<0.001**	1, 375	0.0036	3.21	0.074
	Sex	1, 374	171.31	11.41	**<0.001**	1, 375	0.049	43.85	**<0.001**
	Age	1, 375	102.78	6.85	**0.0092**	1, 374	0.022	19.52	**<0.001**
	Genotype x Treatment	2, 374	12.73	0.42	0.65	2, 374	0.00017	0.076	0.93

Results from the associated type III ANOVA calculated with the Satterthwaite's method on the linear mixed models for standard length and body condition at the three chromosomal inversions. Genotype does not influence standard length or body condition across the three chromosomal inversions. Treatment, sex, and age influence body size measurements across all inversions, except for no effect on body condition from treatment at the chromosome I and XXI inversions. The interaction between treatment and genotype at chromosome I inversion influences body condition. Body condition is the residual of the linear model between standard length and weight. For a visual representation of this data, see Fig. 3. D.F = degrees of freedom; Sum. Sq. = sum of squares. Significant *p*-values (<0.05) are highlighted in bold.

Body condition, which is the residual from the linear regression of weight on standard length, did not differ by inversion genotype at any of the three inversions (Table 2). The interaction between inversion genotype and treatment did not influence body condition for the chromosome XI and XXI inversions. However, a marginal effect of this interaction was observed for chromosome I, where heterozygous individuals had a lower body condition than either homozygous genotype in the freshwater system (Fig. 3). As for standard length, body condition is influenced by age and sex for all inversions; males had a higher body condition (Fig. S1). Unlike for standard length, treatment only had an effect on body condition in crosses segregating the chromosome XI inversion; fish in the freshwater treatment had a higher body condition (Table 2).

## Discussion

Chromosomal inversions are thought to influence adaptation and speciation, yet their fitness effects in natural systems remain poorly understood, limiting our ability to identify the evolutionary factors driving their establishment and spread in populations (Kirkpatrick 2010; Wellenreuther and Bernatchez 2018; Faria et al. 2019; Berdan et al. 2023). To address this knowledge gap, we studied the fitness effects of three chromosomal inversions found in threespine sticklebacks. Specifically, we investigated whether inversion genotypes and salinity treatment influence survival, standard length, and body condition. We found that genotype frequencies across the three chromosomal inversions do not differ from the expected Mendelian ratios in either the freshwater or saltwater treatment. Furthermore, we did not observe an effect of inversion genotype or its interaction with salinity on standard length for any of the three chromosomal inversions. Similarly, there was no effect of genotype at the chromosome XI and XXI inversions or their interaction with salinity on body condition. For the chromosome I inversion, a marginal effect of inversion genotype and its interaction with salinity treatment was observed, where heterozygotes have a lower body condition than both homozygotes in the freshwater treatment. Despite this minor effect, our results mostly indicate that the inversion genotypes do not have strong deleterious effects in either saltwater or freshwater in our laboratory setting. These results further suggest that salinity alone is not a strong selective pressure acting on these inversions. Thus, we propose that if natural selection acts on the inversions, other environmental pressures, either independent of or in combination with salinity, might have caused their spread and maintenance in threespine sticklebacks.

### Absence of lethality or strong deleterious effects of inversion genotype

The absence of lethality or strong deleterious effects associated with the inversions in threespine stickleback contrasts with both theoretical expectations and empirical evidence from other study systems. Evolutionary theory predicts that regions of low recombination, such as inversions, accumulate deleterious mutations (Muller 1932) because of a lower efficacy of selection (Keightley and Otto 2006). A simulation study found that regions associated with inversions degrade in a Muller’s ratchet-like process, which can render one or both orientations inviable (Berdan et al. 2021). Supporting this expectation, several empirical studies report higher mortality in homozygous inverted genotypes or deviations of genotype frequencies associated with the accumulation of mutation load (Wang et al. 2013; Tuttle et al. 2016; Mérot et al. 2018; Jay et al. 2021). An exploration of genotype frequencies at the inversions in threespine sticklebacks has not been done in most natural populations, but freshwater and marine homozygotes can be found at high frequency in the wild (Roesti et al. 2015; Liu et al. 2018), suggesting that neither homozygote genotype is lethal in natural environments. Although individuals that have a homozygous marine genotype at all three inversions can be found at high frequency in freshwater populations (Roesti et al. 2015), individuals with homozygous freshwater genotypes have not been reported in marine populations. Thus, we explicitly tested the viability of the inversion genotypes in both freshwater and saltwater and found that both homozygous genotypes are viable in both salinity treatments. These experimental and natural observations indicate that chromosomal inversions in threespine sticklebacks are not strongly deleterious when homozygous. Several non-mutually exclusive factors may explain this observation, such as the frequency of the inverted orientation, gene conversion, and the methods used to estimate fitness effects.

A balance between selection, drift, and genetic exchange will determine the mutation load associated with an inversion haplotype. Accumulation of deleterious mutations in inversions is limited to stages when they are rare; when inversions are found in a high enough frequency, this effect is expected to be weak (Berdan et al. 2021). Thus, in systems where the inverted or standard orientation is at high frequency, recessive deleterious mutations will be exposed to selection in the homozygous genotype. In agreement with this expectation, a study exploring mutation load in sunflower species found lower load in populations monomorphic for one orientation (Huang et al. 2022). In threespine sticklebacks, both the standard and inverted orientations can be at high or intermediate frequencies (Roesti et al. 2015), which should result in reduced mutation load. In the marine population used in this study, the frequency of the marine orientation of all three inversions is greater than 0.9 (C.L. Peichel, unpublished observation), consistent with the lack of deleterious effect in homozygotes for this orientation. Although the freshwater orientation is at a low frequency in this population, the presence of this orientation in marine populations is thought to result from gene flow with freshwater populations (Colosimo et al. 2005; Jones, Grabherr, et al. 2012; Nelson and Cresko 2018; Bassham et al. 2018). As the freshwater orientation is found at high frequency in freshwater populations, the accumulation of recessive deleterious alleles is not expected. However, measures of mutation load associated with these inversions are needed to directly address this issue.

An additional explanation for the observed lack of deleterious effects in our experimental crosses could also be explained by genetic exchange between the inverted and standard orientations during the evolution of these arrangements. Exchange of genetic material between the different segments is possible through gene conversion, a process in which double-strand breaks are repaired by copying a homologous piece of template DNA (Chovnick 1973). Gene conversion was found as a key factor in determining the mutation load within an inversion, where a higher rate of gene conversion was associated with lower mutation load in both orientations (Berdan et al. 2021). Empirical evidence has shown that inversion heterozygotes experience gene conversion, but this research has been limited to *Drosophila* species (Laayouni et al. 2003; Schaeffer and Anderson 2005; Korunes and Noor 2019; Schaeffer et al. 2024). In threespine sticklebacks, there is ample opportunity for the formation of inversion heterozygotes, suggesting that gene conversion is plausible in this system. Altogether, a combination of factors such as inversion frequency and genetic exchange via gene conversion could explain the lack of strong deleterious effects of chromosomal inversions in sticklebacks. Exploring the allelic content of these chromosomal inversions could help to disentangle these effects and understand the factors that lead to the accumulation of deleterious mutations in regions of reduced recombination.

It is important to note that our estimation of fitness effects associated with the inversions is limited to the fitness proxies of survival, size, and body condition under laboratory conditions within a single generation, which might limit our ability to detect certain fitness effects. Nonetheless, in other systems, inversion genotype has been associated with survival in laboratory conditions (Wang et al. 2013; Tuttle et al. 2016; Jay et al. 2021), as well as body size (Fouet et al. 2012; Kapun et al. 2016; Ayala et al. 2019). In threespine stickleback, body size has often been used as a fitness proxy (Schluter 1994, 1995; Arnegard et al. 2014). Indeed, various measures of body size have been associated with mating success (Nagel and Schluter 1998; Head et al. 2013), clutch size (Baker et al. 2008), reproductive state in females (Bagamian et al. 2004), and the strength of red-throat coloration in males (Barber et al. 2000), a trait associated with reproductive success (Bakker and Milinski 1993). However, we recognize the inherent limitations of using survival, size, and body condition in the laboratory as fitness proxies, and further research is clearly needed to determine whether these inversions are under divergent ecological selection. For example, determining whether inversion genotypes are associated with ecologically-relevant traits in laboratory crosses or specific ecological factors and phenotypes in natural populations will help us to understand whether divergent ecological selection plays a role in the evolution of these chromosomal inversions in threespine sticklebacks. Ultimately, however, direct estimates of the association between inversion genotype and reproductive fitness in natural populations are still needed.

### Environment-specific underdominance in chromosome I heterozygotes?

We observed a reduced body condition for the heterozygotes at chromosome I in the freshwater treatment, which might indicate environment-specific underdominance. Underdominance associated with inversions is commonly thought to result from meiotic problems (Rieseberg 2001), but this should only result in fertility defects that are unlikely to be environment-dependent and that were not measured here. An alternative form of lower heterozygote fitness could result from the interaction of the allelic content in the inversions (Kirkpatrick and Barton 2006). Incompatible alleles could accumulate in inversions because the suppression of recombination between the different orientations would limit the generation of unfit genotype combinations even when populations are parapatric, as is the case for many marine and freshwater stickleback populations (Navarro and Barton 2003; Butlin 2005). Thus, incompatibilities between alleles within the different inversion orientations could explain our observation of lower body condition in heterozygotes, but further exploration is required to test if there are genic incompatibilities associated to the chromosome I inversion and if so, their genetic basis. However, we consider it unlikely that these would be intrinsic Dobzhansky-Muller incompatibilities for two reasons. First, reduced body condition was only observed in the freshwater treatment, suggesting that any such incompatibility would be environmentally dependent. Second, there is no evidence for intrinsic incompatibilities between marine and freshwater sticklebacks (Lackey and Boughman 2017; Thompson et al. 2022).

Although environment-specific underdominance at the chromosome I inversion could explain reduced body condition of heterozygotes in the freshwater treatment, this raises the question of how this inversion could spread in freshwater populations despite this negative fitness effect. However, a key element for the spread of an underdominant inversion is that it also contains alleles that are favored in one environment, suggesting that the chromosome I inversion should contain alleles that are favored in freshwater. One promising candidate gene within this inversion is Na(+)/K(+) transporting ATPase (*Atp1a1a*), a gene involved in osmoregulation (Evans et al. 2005). This gene shows evidence of parallel genomic divergence between marine and freshwater populations across multiple freshwater stickleback radiations (Roesti et al. 2014; Magalhaes et al. 2021) and parallel expression divergence between marine and freshwater populations, with lower expression in freshwater individuals (Verta and Jones 2019). The *Atp1a1a* gene also shows evidence of directional selection and is associated with environmental salinity, indicating that it is likely involved in adaptation to freshwater (Shimada et al. 2011). Furthermore, a study exploring fitness variation in marine- and freshwater-derived individuals grown in saltwater and freshwater treatments found that *Atp1a1a* expression was positively correlated with fitness, particularly in the freshwater treatment (McCairns and Bernatchez 2010). The stronger correlation with fitness in the freshwater treatment is interesting given our observation that the effect of the chromosome I inversion on body condition is limited to the freshwater treatment. Together, these results suggest that the freshwater orientation of the chromosome I inversion could harbor alleles of the *Atp1a1a* gene that are advantageous in freshwater. However, studies exploring whether the sequence and expression differences between marine and freshwater populations in this gene fully co-segregate with the inversion are needed.

### Salinity is not the sole selective pressure at the chromosomal inversions

We found no interaction between salinity treatment and inversion genotype for the chromosome XI and XXI inversions and only a marginal effect of the chromosome I inversion, suggesting that salinity is not a strong selective pressure acting on these inversions. A potential explanation for this observation is that we used a freshwater treatment (3 ppt saltwater) with a higher salinity than what is found in most freshwater habitats. Although this condition was chosen to maximize fish health in our facility (Archambeault et al. 2020), we could be missing effects of the inversions that are only observed in pure freshwater conditions. Nonetheless, we can still conclude that the low frequency of freshwater homozygotes in marine habitats does not result from lethality in high salinity.

In addition, individuals with homozygous marine genotypes at these three inversions can be found at high frequency in freshwater environments (Roesti et al. 2015), further suggesting that the association between marine–freshwater divergence and these inversions is not due solely to salinity. Thus, additional selective pressures beyond or in combination with salinity might be involved in the evolution of these inversions. For example, freshwater populations are not only characterized by low salinity but also experience more variable temperatures than marine populations (Lee and Bell 1999), including temperatures that are much lower than in the ocean (Barrett et al. 2010). A previous study showed that lab-reared individuals from the same marine population used in our study grow more slowly in low salinity than in high salinity, but only at low temperatures of around 4 °C (Gibbons et al. 2017). These results suggest that fitness differences of the inversions might only be expected in combination with variation in temperature, warranting additional research exploring these abiotic factors in combination. Moreover, many biotic factors such as parasites (Weber et al. 2017), predators (Reimchen 1994) and prey (Bell and Foster 1994) differ between marine and freshwater environments, Thus, exploring the association between inversion genotype frequences and ecological variables across the worldwide distribution of stickleback populations will shed additional light on the factors involved in the spread and maintenance of these inversions in nature.

## Conclusions

Despite the increasing number of examples of associations between inversions and ecotype or species divergence, understanding how this form of structural variation evolves and is maintained in natural populations is still a key question in evolutionary biology. Our findings in sticklebacks provide a strong contrast to work in some other systems, in which inversions are associated with intrinsic lethality or the accumulation of strong deleterious mutations. This contrast suggest additional theoretical and empirical work is needed to understand why and when deleterious mutations accumulate within inversions. Furthermore, our study highlights the importance of empirically testing putative sources of selection and fitness consequences of inversions, even when theory and observational data suggest a selective advantage.

## Supplementary information

Supplemental Material

## Data Availability

Phenotype and genotype data, and all scripts used in the analyses are available at 10.5061/dryad.76hdr7t7c.
