# Supplementary material for: Experimental test of the fitness effects of divergent marine–freshwater chromosomal inversions in stickleback under different salinity conditions
Source: Heredity (Edinb). 2025 Jul 24;135(3):152–61. doi: 10.1038/s41437-025-00784-8 (PMC13031518; doi:10.1038/s41437-025-00784-8)
Supplement: Supplementary file 1 — Supplemental Material [file 41437_2025_784_MOESM1_ESM.pdf]

Supplementary Information

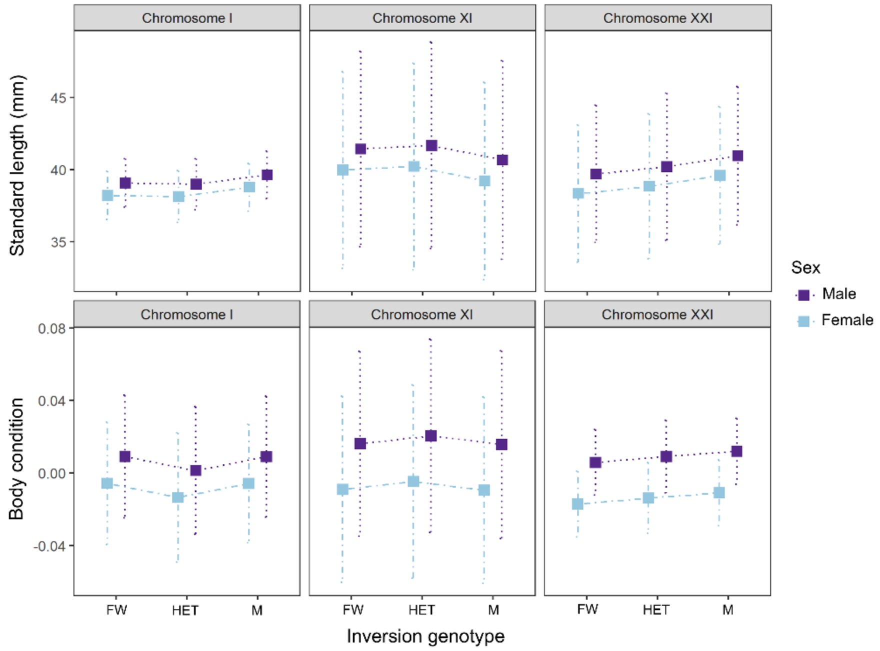

Figure S1. Standard length and body condition across inversion genotypes and sex. The estimated marginal means and 95% confidence intervals for standard length and body condition from linear mixed models are shown across the three chromosomal inversions. FW denotes individuals homozygous for the freshwater-prevalent inversion orientation, HET denotes heterozygous individuals, and M denotes individuals homozygous for the marine-prevalent inversion orientation. Data are jittered within genotype class to better show the confidence intervals for each sex. These results are based on the data across the three families for each inversion and the two salinity treatments.

Table S1. Genotyping primers used in this study. These include the primers needed to distinguish the marine and freshwater alleles across the three chromosomal inversions and the primers to obtain the sex of the individuals.

Genotyping	Marker name	Forward primer (5' – 3')	Reverse primer (3' – 5')	Product size	
				Freshwater	Marine
**Chromosomal inversions**	I	TCAAAGTGAAGCTCCACAGC	GGGTGGAGAGCACAGAGG	242 bp	192 bp
	XI	CACATCAGCCTCGGTAGAGC	TGGTCACATAGTGCCAGTGC	376 bp	325 bp
	XXI	CAGGACCAGTTGTGTTAAATGG	GTACTTGGCTCCTGCTACCG	295 bp	246 bp
				Y	X
**Sexing**	LRR[1]	GGGGAGTGTATGAGTTTAGAGAA	CTCAAAGGCTGAAGGCAGTG	280 bp	550 bp

[1]Archambeault et al., 2020
